# Evaluation of mlstverse system for accurate subspecies identification and drug resistance prediction in *Mycobacterium abscessus* species

**DOI:** 10.1128/spectrum.00643-25

**Published:** 2025-07-31

**Authors:** Wakako Arakaki, Takeshi Kinjo, Wakaki Kami, Hiroe Hashioka, Daijiro Nabeya, Hiroaki Nagano, Shiomi Yoshida, Kazunari Tsuyuguchi, Yuki Matsumoto, Shota Nakamura, Jiro Fujita, Kazuko Yamamoto

**Affiliations:** 1 Department of First Internal Medicine, Division of Infectious, Respiratory, and Digestive Medicine, University of the Ryukyus Graduate School of Medicine 199544 , Okinawa, Japan; 2 Department of Respiratory Medicine, Okinawa Prefectural Chubu Hospital 38506, Okinawa, Japan; 3 Clinical Research Center, NHO Kinki Chuo Chest Medical Center, Osaka, Japan; 4 Department of Infection Metagenomics, Research Institute for Microbial Diseases (RIMD), Osaka University 12936 , Osaka, Japan; 5 Respiratory Medicine, Ohama Dai-ichi Hospital, Okinawa, Japan; Assistance Publique - Hopitaux de Paris Universite Paris Saclay, Clamart, France

**Keywords:** *Mycobacterium abscessus *species, nontuberculous mycobacteria, multilocus sequence typing, mlstverse, subspecies, drug resistance, macrolide, amikacin

## Abstract

Accurate subspecies identification and drug susceptibility testing (DST) are essential for appropriate clinical management, particularly in patients infected with *Mycobacterium abscessus* species (MABS). We developed a novel software, mlstverse, which uses multi-locus sequence typing to identify non-tuberculous mycobacteria (NTM) species, demonstrating rapid and accurate diagnostic performance. However, these studies included only a limited number of MABS samples. In this study, we focused on MABS and evaluated the diagnostic accuracy of the system for subspecies identification and drug resistance prediction for clarithromycin (CAM) and amikacin (AMK). A total of 56 clinical isolates, previously identified as MABS by conventional methods, were analyzed. The mlstverse identified two isolates as *M. intracellulare* and 54 isolates as MABS, with 28 identified as *M. abscessus* subsp. *abscessus* (MAB) and 26 as *M. abscessus* subsp. *massiliense* (MMA). All results obtained through mlstverse were fully consistent with the species/subspecies exhibiting the highest average nucleotide identity (ANI) values. In contrast to the identification by ANI values, the mlstverse system clearly distinguished between MABS subspecies, with the mean differences between the highest and second-highest MLST scores of 0.16 for MAB and 0.33 for MMA, respectively. The system predicted drug susceptibility to CAM and AMK with high concordance to phenotypic DST (CAM: 98.1%, AMK: 100%). These results suggest that mlstverse provides a reliable method for accurate subspecies identification and drug resistance prediction in MABS, supporting the potential of integrating portable next-generation sequencing technologies with real-time software analysis for improved diagnostic accuracy and treatment strategies in patients with NTM infections.

**IMPORTANCE:**

Accurate subspecies identification and drug susceptibility testing (DST) are essential for appropriate clinical management of *Mycobacterium abscessus* species (MABS) infections. We previously developed mlstverse, a novel software utilizing multi-locus sequence typing to identify non-tuberculous mycobacteria (NTM species, demonstrating rapid and accurate diagnostic performance. However, these studies included only a limited number of MABS samples. In this study, we focused on MABS and evaluated the diagnostic accuracy of the system for subspecies identification and drug resistance prediction for clarithromycin (CAM) and amikacin (AMK). We showed that mlstverse can clearly distinguish MAB subspecies compared to ANI values and predicted drug susceptibility to CAM and AMK with high concordance to phenotypic DST. The mlstverse system provides a reliable method for accurate subspecies identification and drug resistance prediction in MABS, supporting the potential of integrating portable next-generation sequencing technologies with real-time software analysis for improved diagnostic accuracy and treatment strategies in patients with NTM infections.

## INTRODUCTION

Non-tuberculous mycobacterial pulmonary disease (NTM-PD) has emerged as a significant global public health issue, responsible for difficult-to-treat respiratory infections that have shown a rising prevalence in recent years (1, 2). With approximately 200 species of NTM, each exhibiting different pathogenicity, drug susceptibility, and prognosis, the accurate identification of the causative NTM species is essential, both for clinical practice and for understanding of the epidemiology. *Mycobacterium abscessus* species (MABS), which causes refractory NTM-PD with limited chemotherapeutic options due to both intrinsic and acquired drug resistance, comprises three subspecies—*M. abscessus* subsp. *abscessus* (MAB), *M. abscessus* subsp. *massiliense* (MMA), and *M. abscessus* subsp. *bolletii* (MBO)—each exhibiting distinct bacteriological characteristic (3). For example, the survival rate in untreated NTM-PD patients with MAB was reported to be higher than those with MMA, suggesting differences in pathogenicity between MAB and MMA (4). In terms of drug susceptibility, MAB and MBO have functional *erm*(41) genes which induce resistance to macrolide antibiotics, whereas this gene is truncated in MMA, thus susceptible to macrolides (5). Although this drug susceptibility pattern may occasionally alter by mutations in the *erm*(41) or *rrl* genes (6), the identification of MABS to the subspecies level is crucial for clinical practice.

Recently, matrix-assisted laser desorption ionization-time of flight mass spectrometry (MALDI-TOF MS) has been widely used for NTM species identification. However, MALDI-TOF MS cannot distinguish closely related species nor identify subspecies, and it has been reported that approximately 30% of NTM isolates cannot be identified even in the species level (7, 8). In terms of drug susceptibility testing (DST), the broth microdilution method has been used as a gold standard method. However, its limitation includes variations in result interpretation between institutions or laboratory technicians, the need for specialized procedural skills, risk of contamination, and long turnaround time (9, 10). Furthermore, the test may be infeasible if the bacteria exhibit poor growth on the microplate.

Against this background, there is a need for the development of a diagnostic system capable of accurately and comprehensively identifying NTM at the subspecies level while simultaneously evaluating drug susceptibility. With the advancement of next-generation sequencing (NGS) technology, portable NGS devices facilitate the real-time evaluation and analysis of sequencing data. Furthermore, through integration with analytical software, point-of-care diagnostics for bacterial pathogens using NGS is progressively becoming a feasible reality (11). In a previous study, we developed a novel software, mlstverse, which can comprehensively identify NTM species based on multi-locus sequence typing (MLST) targeting 184 genes (12). The process, encompassing base calling, database mapping, calculating MLST score, and score filtering, is already configured for automatic execution during sequencing, and the identified species name is shown on the screen via the in-house website in around 10 min. This test can be performed at any location with an internet-connected laptop and a portable NGS device within a standard laboratory setting, thus requiring no specialized equipment. Recently, Fukushima et al. demonstrated the efficacy of the system in predicting drug resistance in addition to identifying NTM species (13). However, these studies included only a few MABS samples. In this study, we focused on MABS and evaluated the diagnostic accuracy of the system for subspecies identification and drug resistance prediction for clarithromycin (CAM) and amikacin (AMK), key agents for MABS treatment, using sufficient sample size.

## MATERIALS AND METHODS

### Samples

A total of 56 clinical isolates (49 from sputum, one from blood, and six from skin pus), previously identified as MABS by either DNA-DNA hybridization (DDH mycobacteria ’KYOKUTO’, Kyokuto Pharmaceutical Industrial, Tokyo, Japan) or MALDI-TOF MS (VITEK MS V3.2, bioMérieux, Marcy-l'Étoile, France) at the University of the Ryukyus Hospital and its affiliated hospitals in Okinawa, Japan, between January 2009 and September 2022, were used in this study. These isolates, stored in 10% skim milk at deep freezer (−80°C), were cultured with 2% Ogawa medium (Kyokuto Pharmaceutical Industrial, Tokyo, Japan) at 30°C for 3–5 days, and then used for DST and genetic analysis.

### Phenotypic drug susceptibility testing (DST)

Phenotypic DST was conducted using the broth microdilution method (BrothMIC RGM, Kyokuto Pharmaceutical Industrial, Tokyo, Japan), which complied with the 3rd Ed, Clinical Laboratory Standards Institute (CLSI) criteria (14), following the manufacturer’s instructions. Briefly, the bacterial colonies were dissolved with distilled water and the turbidity was adjusted to 0.5 McFarland equivalent. Then, 50 µL of the solution was added to cation-adjusted Mueller-Hinton broth provided in the product. Finally, 100 µL of the solution was inoculated into each well on the microplate and cultured at 30°C. Drug susceptibility was determined according to CLSI criteria (14) based on the results of minimum inhibitory concentration (MIC).

### Genomic analysis for subspecies identification

Qiagen DNeasy PowerSoil Pro Kit (QIAGEN, Hilden, Germany) was used for DNA extraction. The purity of the isolated DNA A260/A280 ratio was measured by UV spectrophotometry (Nanodrop Technologies, Wilmington, CA). The extracted DNA was processed for library preparation following the standard protocols for Ligation Sequencing Kit (SQK-LSK109, Oxford Nanopore Technologies, Oxford, UK). The steps involved DNA fragmentation, end repair, adapter ligation, and purification. Sequencing runs were performed using the MinION instrument (MIN-101B, Oxford Nanopore Technologies, Oxford, UK). Identification of NTM species was performed by mlstverse software, as described in the previous article (12). After mapping raw sequencing reads to the sequences of loci using minimap2 (15), MLST scores, ranging between 0 and 1, were calculated using mlstverse database, which contains 1,398 profiles of MABS subspecies (MAB: 912, MAS: 379, MBO: 107). The MLST score for each NTM species/subspecies was displayed graphically over time on the computer, with the species/subspecies exhibiting the highest MLST score being determined as the identified one. The heat map image of MLST scores for all profiles of MABS subspecies in each sample was visualized using a custom R script. The average nucleotide identity (ANI) values for each strain were calculated using fastANI from assembly sequences obtained with Flye, comparing them with representative genomic sequences of MABS subspecies as references (16, 17). The RefSeq accession numbers were GCF_900141695.1 for MAB, GCF_000497265.2 for MAM, and GCF_001792615.1 for MBO.

### Genomic analysis for drug susceptibility prediction

In addition to NTM identification, we incorporated a system into mlstverse to enable the simultaneous prediction of drug susceptibility to CAM and AMK by analyzing the responsible genes. Regarding CAM, mutations in *rrl* (positions 2058 and 2059 [*Escherichia coli* numbering] in 23S rRNA) and *erm*(41) (T-to-C substitution at position 28 [T28C] and truncation) were analyzed, and the system displayed either “susceptible,” “inducible resistant,” or “acquired resistant” based on the pattern of gene mutations (Table S1). For AMK, mutations in *rrs* (A1408G, C1409T, or T1498A [*E. coli* numbering] in 16S rRNA) were analyzed, and the system displayed either “susceptible” or “resistant” based on the presence of the above mutations. Concordance rates were calculated by comparing results between genetic prediction and phenotypic DST.

### Statistical analysis

The Steel test was used to compare MLST scores between the control group (identified subspecies) and comparative groups (the other two subspecies) by JMP Pro 15 (SAS Institute Inc., USA). A *P*-value of <0.05 was considered statistically significant.

## RESULT

### MABS subspecies identification

**Fig 1:**
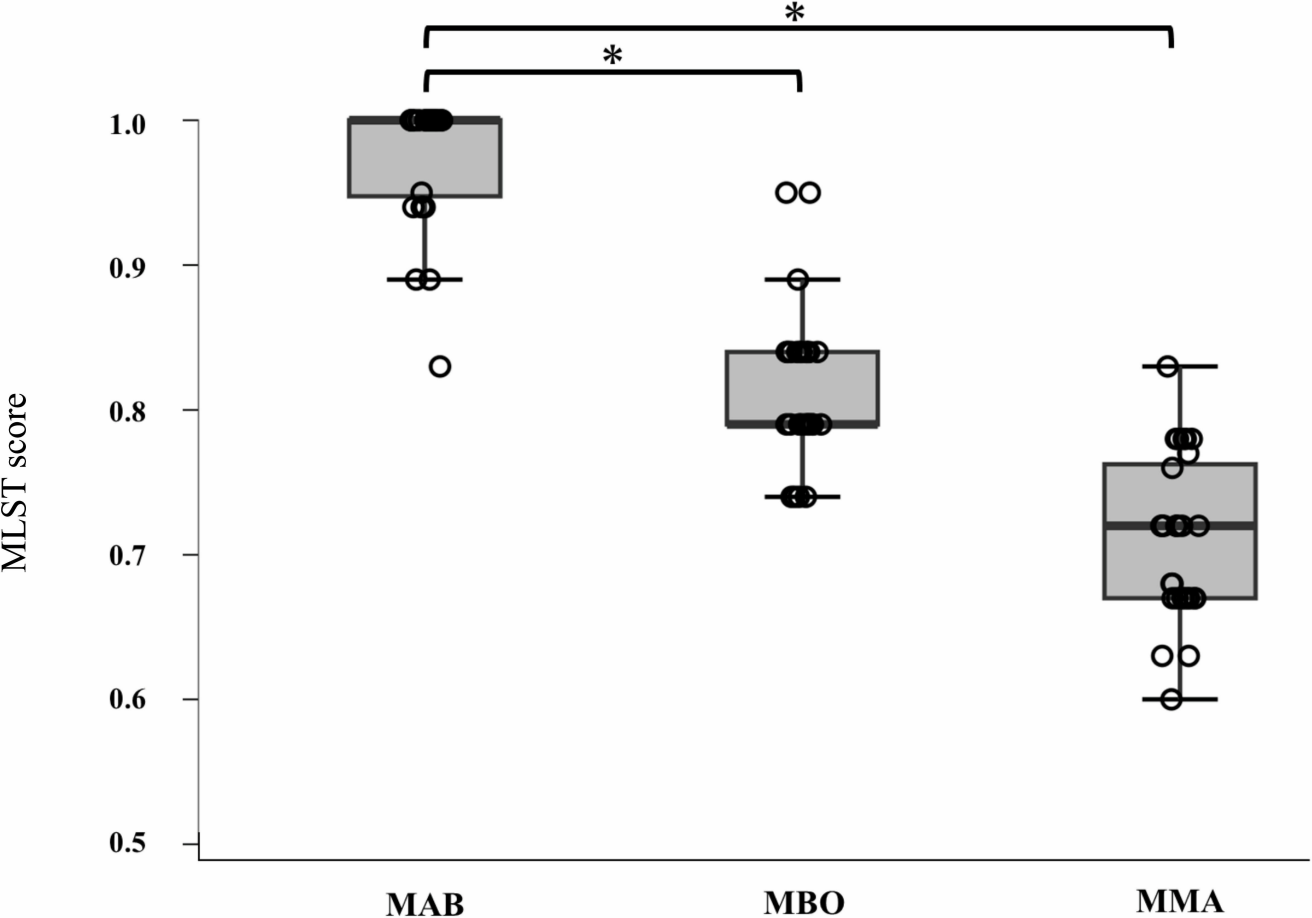
MLST scores for three subspecies in MAB samples. MLST scores for three subspecies were compared by the Steel test. *P < 0.001. MAB, *Mycobacterium abscessus* subsp. *abscessus*; MBO, *Mycobacterium abscessus* subsp. *bolletii*; MMA, *Mycobacterium abscessus* subsp. *massiliense.*

**Fig 2:**
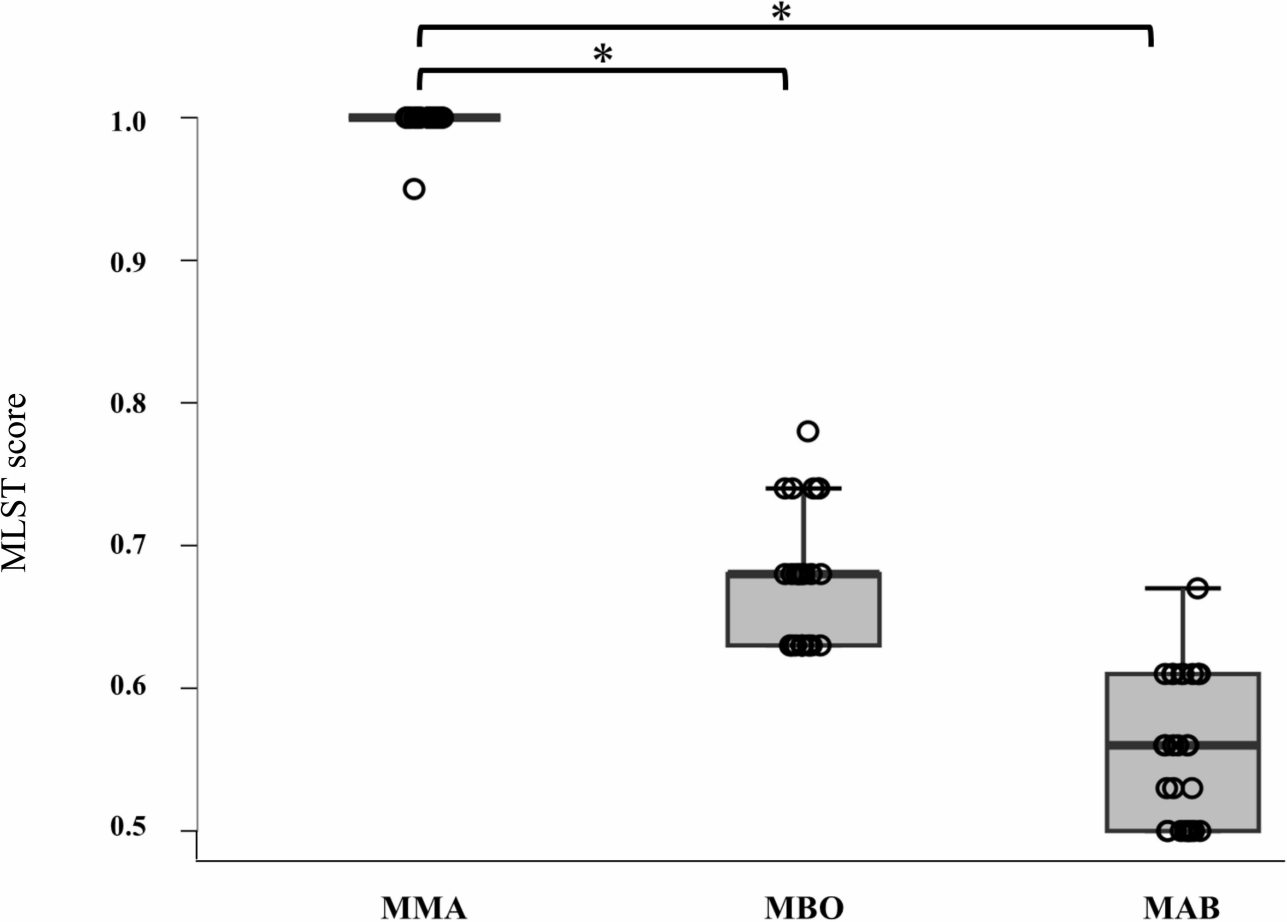
MLST scores for three subspecies in MMA samples. MLST scores for three subspecies were compared by the Steel test. *P < 0.001. MAB*, Mycobacterium abscessus* subsp. *abscessus*; MBO, *Mycobacterium abscessus* subsp. *bolletii*; MMA, *Mycobacterium abscessus* subsp. *massiliense*

**Fig 3:**
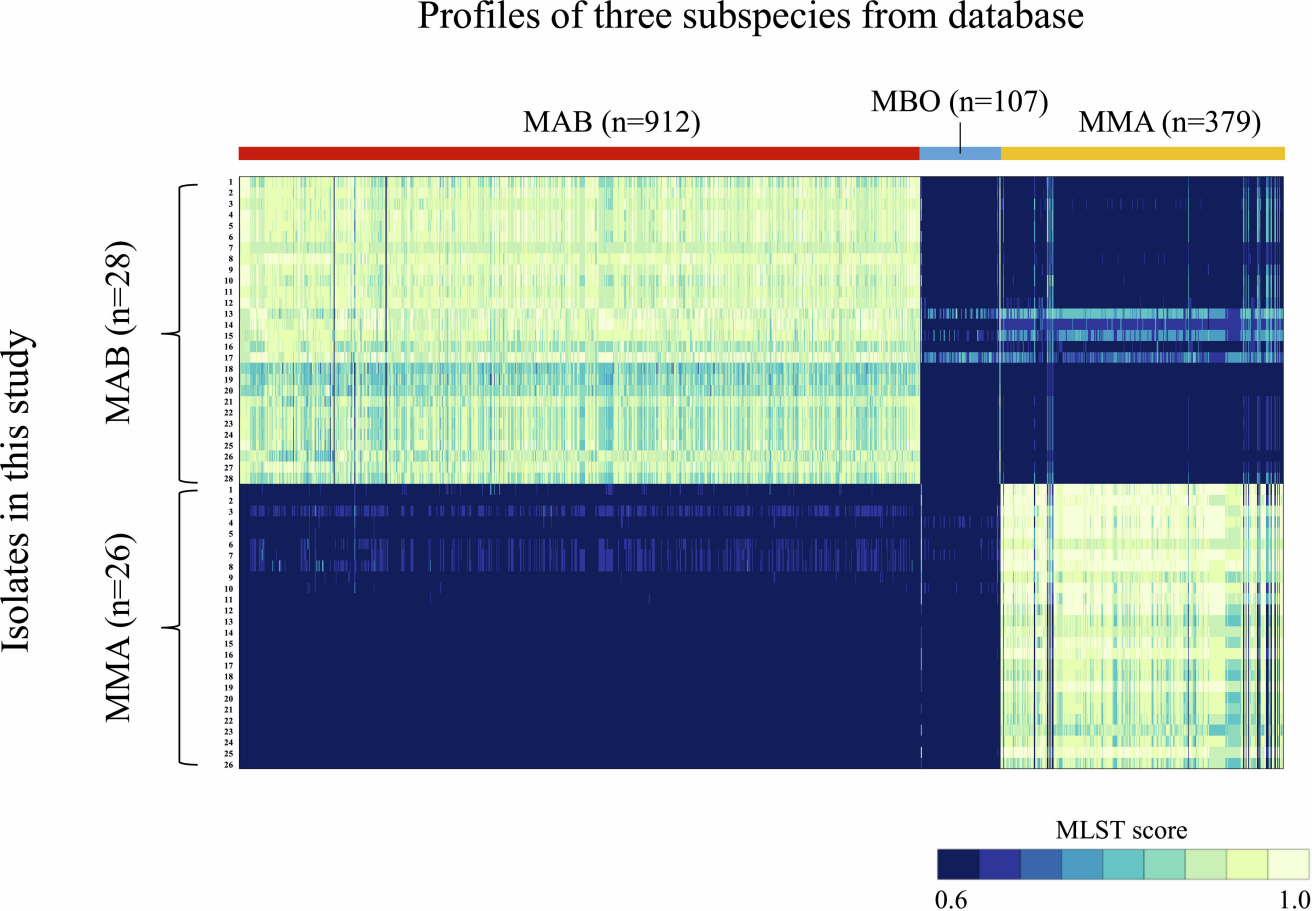
Heat map image. The heat map image of MLST scores for all registered profiles of MABS subspecies in each sample was visualized using a custom R script.

The mlstverse identified 54 isolates as MABS and two isolates as *M. intracellulare*, with all results obtained by mlstverse fully corresponding with the species/subspecies showing the highest ANI value (Table S2 and S3). Of the 54 MABS isolates, 28 and 26 strains were identified as MAB and MMA, respectively. To evaluate the discrimination ability of MABS subspecies identification, MLST scores of the identified subspecies were compared with those of the other two subspecies. As for 28 MAB strains, the mean MLST scores were 0.98 (range, 0.89–1.00) for MAB, 0.82 (range, 0.74–0.95) for MBO, and 0.71 (range, 0.60–0.83) for MMA, respectively (Table S2). As shown in Fig. 1, the MLST score for MAB was significantly higher than MBO and MMA (*P* < 0.001), and the mean difference between the highest and second-highest MLST scores was 0.16 (range, 0.05–0.26). In terms of 26 MMA strains, the mean MLST scores were 0.99 (range, 0.95–1.00) for MMA, 0.67 (range, 0.63–0.78) for MBO, and 0.56 (range, 0.50–0.67) for MAB, respectively (Table S3). As shown in Fig. 2, the MLST score for MMA was significantly higher than MBO and MAB (*P* < 0.001), and the mean difference between the highest and second-highest MLST scores was 0.33 (range, 0.21–0.37). The mean differences between the highest and second-highest ANI values for MAB and MMA (Table S2 and S3) were calculated to be 2.0% and 1.5%, respectively. These results showed the mlstverse system clearly distinguished MABS subspecies. The discrimination ability of mlstverse can also be visually confirmed by the heatmap image (Fig. 3).

### Phenotypic DST

Tables 1 and 2 show the MIC distributions of MAB and MMA, respectively. One MAB strain was undeterminable because of its poor growth on the broth microdilution plate. Regarding CAM susceptibility for MAB, 10 strains were determined to be susceptible, while 16 strains exhibited inducible resistance. One strain demonstrated an MIC of 8 µg/mL on day 3, indicating acquired resistance according to the CLSI criteria (Table 1). For MMA, all 26 strains were determined to be CAM-susceptible (Table 2). The MIC of AMK for both MAB and MMA was less than 32 on day 3, indicating susceptibility to AMK.

**TABLE 1:** MIC distribution for MAB (*n* = 27)^*a*^^,^^*b*^

Antibiotics	Number of isolates with MIC (μg/mL) of:													
	<0.06	0.06	0.12	0.2	0.5	1	2	4	8	16	32	64	>64	>152/8
CAM (3 days)	0	0	7	4	4	5	3	3	1	0	0	0	0	
CAM (14 days)	0	0	0	6	4	0	0	0	0	1	0	0	16	
AZM (3 days)	0	0	0	0	4	6	3	4	0	4	2	1	3	
AZM (14 days)	0	0	0	0	0	2	5	3	0	0	0	0	17	
AMK	0	0	0	0	0	0	0	3	18	4	2	0	0	
TOB	0	0	0	0	0	0	1	6	12	7	1	0	0	
IPM	0	0	0	0	0	0	0	0	16	9	2	0	0	
MEPM	0	0	0	0	0	0	0	0	0	0	12	12	3	
FRPM	0	0	0	0	0	0	0	0	0	0	0	0	27	
LVFX	0	0	0	0	0	0	0	0	0	12	4	11	0	
STFX	0	0	0	0	0	9	15	3	0	0	0	0	0	
MFLX	0	0	0	0	0	0	0	4	4	19	0	0	0	
DOXY	0	0	0	0	0	0	0	0	0	0	27	0	0	
LZD	0	0	0	0	0	0	0	0	4	2	11	10	0	
CLF	0	0	0	0	18	8	1	0	0	0	0	0	0	
ST														27

^*a*^ Abbreviations: AMK, amikacin; AZM, azithromycin; CAM, clarithromycin; CLF, clofazimine; DOXY, doxycycline; FRPM, faropenem; IPM, imipenem; LVFX, levofloxacin; LZD, linezolid; MAB, *Mycobacterium abscessus* subsp. *abscessus*; MEPM, meropenem; MFLX, moxifloxacin; MIC, minimum inhibitory concentration; ST, sulfamethoxazole/trimethoprim; STFX, sitafloxacin; TOB, tobramycin.

^*b*^ Empty cells indicates that these MICs were not included as measurement items.

**TABLE 2:** MIC distribution for MMA (*n* = 26)^*a*^^,^^*b*^

Antibiotics	Number of isolates with MIC (μg/mL) of:														
	<0.06	0.06	0.12	0.2	0.5	1	2	4	8	16	32	64	>64	>76/4	>152/8
CAM (3 days)	0	1	0	6	19	0	0	0	0	0	0	0	0		
CAM (14 days)	0	0	0	6	15	5	0	0	0	0	0	0	0		
AZM (3 days)	0	0	0	0	7	4	12	3	0	0	0	0	0		
AZM (14 days)	0	0	0	0	4	3	9	10	0	0	0	0	0		
AMK	0	0	0	0	0	0	0	4	21	1	0	0	0		
TOB	0	0	0	0	0	0	0	3	8	15	0	0	0		
IPM	0	0	0	0	0	0	0	1	12	9	3	1	0		
MEPM	0	0	0	0	0	0	0	0	0	1	4	9	12		
FRPM	0	0	0	0	0	0	0	0	0	0	0	1	25		
LVFX	0	0	0	0	0	0	0	0	4	4	9	9	0		
STFX	0	0	0	0	3	3	12	8	0	0	0	0	0		
MFLX	0	0	0	0	0	0	2	4	3	17	0	0	0		
DOXY	0	0	0	0	0	0	0	0	1	0	25	0	0		
LZD	0	0	0	0	0	0	2	1	2	4	8	9	0		
CLF	0	0	0	0	18	8	0	0	0	0	0	0	0		
ST														2	24

^*a*^ Abbreviations: AMK, amikacin; AZM, azithromycin; CAM, clarithromycin; CLF, clofazimine; DOXY, doxycycline; FRPM, faropenem; IPM, imipenem; LVFX, levofloxacin; LZD, linezolid; MEPM, meropenem; MFLX, moxifloxacin; MIC, minimum inhibitory concentration; MMA, *Mycobacterium abscessus* subsp. *massiliense*; ST, sulfamethoxazole/trimethoprim; STFX, sitafloxacin; TOB, tobramycin.

^*b*^ Empty cells indicates that these MICs were not included as measurement items.

### Genetic prediction of drug susceptibility

To evaluate the ability of mlstverse in predicting drug susceptibility, the concordance rate with phenotypic DST was examined. One MAB strain with undeterminable phenotypic DST results was excluded from the analysis, leaving a total of 27 MAB strains for comparison. In terms of CAM susceptibility (Table 3), 10 strains were predicted as “susceptible” because T28C mutation on *erm*(41) was detected without *rrl* mutation. Seventeen strains were predicted as “induced resistant” because T28C and *rrl* mutations were absent. Regarding MMA, all 26 strains had truncation of the *erm*(41) gene without *rrl* mutation, thus predicted as “susceptible” (Table 3). These predictions were completely matched with the results by phenotypic DST except for one MAB strain. This strain, genetically predicted as “induced resistant,” was determined to be acquired resistance by phenotypic DST; thus, these results were unmatched. As a result, the concordance rate between phenotypic DST and genetic prediction for CAM susceptibility was 96.3% (26/27) and 100% (26/26) for MAB and MMA, respectively. In total, the concordance rate reached 98.1% (52/53). For AMK susceptibility (Table 4), mutations of A1408G, C1409T, and T1498A were not detected in all MAB and MMA strains, thus predicted as “susceptible.” Since DST results showed all strains were AMK-susceptible, genetic prediction was completely matched with the phenotypic DST results.

**TABLE 3:** Accuracy of mlstverse for CAM susceptibility prediction^*a*^

Subspecies	DST (phenotypic)		Mlstverse (genetic prediction)				Concordance rate (%)	
	Result	N	23S rRNA (*Rrl*)	*Erm*(41)		Result		
			A2058/A2059 [GC]	T28C	Truncation			
MAB	S	10	0/10	10/10	0/10	S	96.3% (26/27)	98.1% (52/53)
	IR	16	0/16	0/16	0/16	IR		
	AR	1	0/1	0/1	0/1	IR		
MMA	S	26	0/26	0/26	26/26	S	100% (26/26)	

^*a*^ Abbreviations: AR, acquired resistant; CAM, clarithromycin; DST, drug susceptibility testing; IR, induced resistant; MAB, *Mycobacterium abscessus* subsp. *abscessus*; MMA, *Mycobacterium abscessus* subsp. *massiliense*; S, susceptible.

**TABLE 4:** Accuracy of mlstverse for AMK susceptibility prediction^*a*^

Subspecies	DST (phenotypic)		Mlstverse (genetic prediction)				Concordance rate (%)
	Result	N	16S rRNA (rrs)			Result	
			A1408G	C1409T	T1498A		
MAB	S	27	0/27	0/27	0/27	S	100% (53/53)
MMA	S	26	0/26	0/26	0/26	S	

^*a*^ Abbreviations: AMK, amikacin; DST, drug susceptibility testing; MAB, *Mycobacterium abscessus* subsp. *abscessus*; MMA, *Mycobacterium abscessus* subsp. *massiliense*; S, susceptible.

## DISCUSSION

We previously developed the mlstverse software, which enables the comprehensive identification of NTM species (12). However, the diagnostic accuracy for the subspecies identification and genetic prediction of drug resistance for MABS has not been sufficiently investigated. This study shows that mlstverse can clearly distinguish MABS subspecies and correctly predict drug susceptibility to CAM and AMK.

MALDI-TOF MS is a widely used method for bacterial identification. It offers advantages, such as cost-effectiveness and rapid results, as it can be applied directly to cultured specimens. In recent years, the expansion of the database has also increased the number of NTM that can be identified, and in fact, Bruker Biotyper can accurately identify 314 strains or 73 species of NTM (18). However, since MALDI-TOF MS identifies bacterial species by ionizing ribosome-derived proteins and measuring their energy peaks using a pulsed laser, the identification of closely related species and subspecies with high sequence similarity in 16S rRNA region tends to be more difficult (19–21). Therefore, accurate identification to the subspecies level by MALDI-TOF MS is technically difficult. Polymerase chain reaction (PCR)-based method has been conventionally used to identify MABS subspecies. Although past studies showed that MABS subspecies can be distinguished by using several housekeeping genes, such as *rpoB*, *hsp65*, and *secA1*, this method is labor-intensive, time-consuming, and requires expert interpretation (22, 23). Recently, commercially available PCR-based kits are available; however, they can detect a narrow range of NTM species with limited sensitivity and specificity (24, 25). In principle, the PCR method can only detect the target bacterial species, and it cannot provide a comprehensive identification of NTM at the subspecies level.

In recent years, the use of NGS has facilitated the accurate identification of bacterial species (11, 24). NGS enables us to acquire a large amount of genomic information comprehensively and simultaneously, allowing for highly accurate identification of bacterial species. Additionally, advancements in NGS technology and the integration of analytical software are making point-of-care diagnostics for bacterial pathogens increasingly feasible through portable NGS devices. We developed mlstverse, a novel analytical software that identifies NTM species using MLST targeting 184 genes and can be run with an internet-connected laptop and portable NGS device, yielding results in about 10 min without specialized equipment (12). However, the performance of subspecies identification for MABS, a pathogen for which subspecies identification is clinically important, has not been sufficiently evaluated. In this study, we demonstrated that the mlstverse system clearly distinguishes between MABS subspecies. To evaluate the discrimination ability, the mean difference between the highest and second-highest MLST scores was calculated, with mean differences of 0.16 for MAB and 0.33 for MMA. The ANI values among MABS subspecies have been reported to range from 96.6% to 97.6% (26). Consistently, the 54 MABS strains used in this study exhibited identities within a similar range of 96.5% to 97.4% when evaluated by ANI (Table S2 and S3). The mean differences between the highest and second-highest ANI values for MAB and MMA were calculated to be 2.0% and 1.5%, respectively, indicating that these differences were substantially smaller compared with those obtained by mlstverse (16% for MAB and 33% for MMA). These results demonstrate that the mlstverse system, which employs MLST method targeting 184 genes, can clearly distinguish MAB subspecies with sufficient accuracy.

Macrolides are key agents in the treatment of MABS infections; however, resistance may develop through two distinct mechanisms: acquired and inducible resistance (27). According to the British Thoracic Society guideline, differentiation between these two forms of macrolide resistance is recommended, as each requires a distinct therapeutic strategy (28). In phenotypic DST, acquired and inducible resistance are determined by evaluating MICs on days 3 and 14, respectively. However, phenotypic DST has several limitations, including variability in result interpretation, the requirement for specialized expertize, prolonged turnaround time, risk of contamination, and potential infeasibility with poor bacterial growth (9, 10). In fact, DST for one MAB strain was infeasible due to poor growth on the broth microdilution plate, providing a concrete example of the limitation of broth microdilution method.

Given this background, the prediction of drug resistance by analyzing genes responsible for drug resistance is beneficial in clinical practice. Acquired resistance to macrolides in MABS is primarily attributed to mutations in the *rrl* gene. Notably, 98.5% of MABS exhibiting acquired resistance harbor mutations at positions 2,058 or 2,059 of the *rrl* gene (29). These mutations have been reported to confer high-level acquired resistance to CAM (MIC of >64 µg/mL at day 3) (30, 31). Mutations at alternative sites within the *rrl* gene may also confer macrolide resistance; however, such mutations are typically associated with low-level resistance (32). MABS harboring the *erm*(41) T28 sequevar exhibit inducible resistance to macrolides, whereas truncation or a T28C mutation in the *erm*(41) gene renders them susceptible (33). The T28C mutation results in an amino acid substitution (Trp to Arg) and functionally inactivates the *erm*(41) gene. Although an exception has been reported (34), mutations at other positions in *erm*(41) generally have minimal impact on the protein structure and thus preserve its function in conferring inducible resistance to macrolides (35, 36). As previously described (27), the selection of the gene set for predicting macrolide resistance in this study was considered an appropriate and rational approach.

In this study, one MAB strain, identified as having acquired resistance to CAM based on phenotypic DST (MIC of 8 µg/mL on day 3), did not exhibit mutations at either position 2058 or 2059. Therefore, there was an inconsistency between the phenotypic DST results and genetic predictions. We further investigated mutations at positions 2,281 and 2,293, both of which have been implicated in acquired resistance to CAM (37). However, no mutations were detected at either position. It has been noted that strains with inducible resistance may be misinterpreted as having acquired resistance if their growth rate in broth is rapid (38). Indeed, it has been reported that inducible resistant strains lacking *rrl* gene mutations may exhibit MIC values of ≥8 µg/mL on day 3, leading to misinterpretation as acquired resistance (27). The prediction of drug susceptibility through genomic analysis could serve as a complementary approach to address the limitations of DST, thereby providing valuable information for clinical practice.

There are several limitations of this study. First, this study was a retrospective, single-center study. Additional evaluation using strains isolated from multiple regions or countries, rather than a specific region, would be desirable. Second, MBO was not included in this study. Therefore, it is essential to evaluate MBO strains; however, due to the rare detection of MBO in NTM-PD patients, the collection of MBO strains remains challenging. Finally, clinical isolates harboring *rrl* or *rrs* mutations were not included in our sample set. We additionally evaluated a clinical isolate of MMA having resistance to AMK (MIC > 16) and confirmed that our system correctly detected the A1408G mutation (data not shown). The frequency of *rrl* and *rrs* mutations among MABS in Japan was reported to be 3.2% and 1.3%, respectively (39, 40). Although the collection of MABS isolates with these mutations is challenging, further evaluation with a larger number of drug-resistant isolates is warranted.

In conclusion, comprehensive subspecies-level identification, particularly for MABS, along with simultaneous prediction of drug resistance to CAM and AMK using the mlstverse system, can be conducted within a standard laboratory setting, making it a promising tool for clinical practice.

## Data Availability

The data sets supporting the conclusions of this study are included in this article. The data sets generated and analyzed here are available from the corresponding author upon reasonable request. The raw sequencing data supporting the findings of this study have been deposited in the NCBI’s SRA under BioProject PRJNA1276484.

## SUPPLEMENTAL MATERIAL

Supplemental tables spectrum.00643-25-s0001.docx Tables S1 to S3.

ASM does not own the copyrights to Supplemental Material that may be linked to, or accessed through, an article. The authors have granted ASM a non-exclusive, world-wide license to publish the Supplemental Material files. Please contact the corresponding author directly for reuse.
