# Supplementary material for: Evaluation of mlstverse system for accurate subspecies identification and drug resistance prediction in Mycobacterium abscessus species
Source: Microbiol Spectr. 2025 Jul 31;13(9):e00643-25. doi: 10.1128/spectrum.00643-25 (PMC12403713; doi:10.1128/spectrum.00643-25)
Supplement: Supplemental tables — Tables S1 to S3. [file spectrum.00643-25-s0001.docx]

Supplementary Table1. Pattern of gene mutation and the interpretation by mlstverse

| 23S rRNA (*rrl*) | *erm*(41) | | mlstverse |
| --- | --- | --- | --- |
| A2058/A2059 [GC] | T28C | Truncation |  |
| + | any | | AR |
| - | - | - | IR |
| - | + | - | S |
| - | - | + | S |

Abbreviations: AR; acquired resistant, IR; induced resistant, S; susceptible

Supplementary Table 2. MLST scores and ANI values for MAB

| No. | MLST score | | | Minimum Difference* |  | ANI | | | Minimum Difference** (%) |
| --- | --- | --- | --- | --- | --- | --- | --- | --- | --- |
|  | MAB | MMA | MBO |  |  | MAB | MMA | MBO |  |
| 1 | 1 | 0.76 | 0.79 | 0.21 |  | 99.5 | 97.1 | 96.9 | 2.43 |
| 2 | 1 | 0.78 | 0.84 | 0.16 |  | 99.4 | 97.0 | 96.8 | 2.36 |
| 3 | 1 | 0.83 | 0.79 | 0.17 |  | 98.8 | 96.9 | 96.9 | 1.94 |
| 4 | 1 | 0.72 | 0.79 | 0.21 |  | 99.2 | 97.0 | 96.8 | 2.16 |
| 5 | 1 | 0.78 | 0.84 | 0.16 |  | 99.5 | 97.1 | 96.9 | 2.44 |
| 6 | 1 | 0.78 | 0.84 | 0.16 |  | 99.5 | 97.1 | 96.9 | 2.42 |
| 7 | 0.94 | 0.72 | 0.84 | 0.1 |  | 98.8 | 96.9 | 96.8 | 1.90 |
| 8 | 1 | 0.72 | 0.79 | 0.21 |  | 98.9 | 97.1 | 96.9 | 1.86 |
| 9 | 1 | 0.68 | 0.79 | 0.21 |  | 99.6 | 97.1 | 96.9 | 2.44 |
| 10 | 1 | 0.72 | 0.84 | 0.16 |  | 98.9 | 97.4 | 97.0 | 1.50 |
| 11 | 1 | 0.77 | 0.79 | 0.21 |  | 99.3 | 97.1 | 96.9 | 2.21 |
| 12 | 1 | 0.67 | 0.84 | 0.16 |  | 99.5 | 97.1 | 96.9 | 2.49 |
| 13 | 1 | 0.78 | 0.95 | 0.05 |  | 98.8 | 97.0 | 96.9 | 1.83 |
| 14 | 0.89 | 0.6 | 0.79 | 0.1 |  | 98.9 | 97.0 | 96.8 | 1.89 |
| 15 | 0.83 | 0.67 | 0.74 | 0.09 |  | 98.9 | 97.1 | 96.9 | 1.88 |
| 16 | 1 | 0.72 | 0.74 | 0.26 |  | 98.9 | 97.2 | 96.8 | 1.70 |
| 17 | 0.94 | 0.67 | 0.84 | 0.1 |  | 98.9 | 97.0 | 96.8 | 1.90 |
| 18 | 0.94 | 0.67 | 0.74 | 0.2 |  | 99.1 | 97.1 | 97.1 | 1.96 |
| 19 | 0.94 | 0.67 | 0.79 | 0.15 |  | 99.2 | 97.2 | 97.2 | 1.94 |
| 20 | 0.89 | 0.67 | 0.74 | 0.15 |  | 99.1 | 97.2 | 97.1 | 1.96 |
| 21 | 1 | 0.72 | 0.89 | 0.11 |  | 99.2 | 97.2 | 97.1 | 1.99 |
| 22 | 1 | 0.72 | 0.84 | 0.16 |  | 99.0 | 97.3 | 97.0 | 1.74 |
| 23 | 1 | 0.63 | 0.79 | 0.21 |  | 99.3 | 97.2 | 97.1 | 2.08 |
| 24 | 1 | 0.67 | 0.79 | 0.21 |  | 99.0 | 97.1 | 97.0 | 1.83 |
| 25 | 1 | 0.63 | 0.79 | 0.21 |  | 99.6 | 97.2 | 96.9 | 2.43 |
| 26 | 1 | 0.67 | 0.95 | 0.05 |  | 99.0 | 97.1 | 97.0 | 1.87 |
| 27 | 0.95 | 0.68 | 0.84 | 0.11 |  | 99.0 | 97.2 | 97.0 | 1.79 |
| 28 | 1 | 0.78 | 0.84 | 0.16 |  | 99.2 | 97.2 | 97.0 | 1.99 |

*Difference between the highest and second-highest MLST scores

**Difference between the highest and second-highest ANI values

Supplementary Table 3. MLST scores and ANI values for MMA

| No. | MLST score | | | Minimum Difference* |  | ANI | | | Minimum Difference** (%) |
| --- | --- | --- | --- | --- | --- | --- | --- | --- | --- |
|  | MAB | MMA | MBO |  |  | MAB | MMA | MBO |  |
| 1 | 0.61 | 1 | 0.63 | 0.37 |  | 97.1 | 98.3 | 96.6 | 1.20 |
| 2 | 0.61 | 1 | 0.74 | 0.26 |  | 96.9 | 98.6 | 96.6 | 1.72 |
| 3 | 0.61 | 1 | 0.63 | 0.37 |  | 96.7 | 98.4 | 96.5 | 1.75 |
| 4 | 0.61 | 1 | 0.63 | 0.37 |  | 97.1 | 98.3 | 96.6 | 1.20 |
| 5 | 0.61 | 1 | 0.68 | 0.32 |  | 97.1 | 98.4 | 96.6 | 1.29 |
| 6 | 0.61 | 1 | 0.78 | 0.22 |  | 97.1 | 98.4 | 96.6 | 1.23 |
| 7 | 0.67 | 1 | 0.74 | 0.26 |  | 96.9 | 98.5 | 96.5 | 1.56 |
| 8 | 0.5 | 1 | 0.68 | 0.32 |  | 96.8 | 99.6 | 96.9 | 2.69 |
| 9 | 0.61 | 1 | 0.68 | 0.32 |  | 97.2 | 98.4 | 96.7 | 1.23 |
| 10 | 0.61 | 1 | 0.68 | 0.32 |  | 97.2 | 98.4 | 96.7 | 1.24 |
| 11 | 0.56 | 1 | 0.68 | 0.32 |  | 97.0 | 98.6 | 96.6 | 1.60 |
| 12 | 0.5 | 1 | 0.63 | 0.37 |  | 97.2 | 98.5 | 96.7 | 1.26 |
| 13 | 0.5 | 1 | 0.63 | 0.37 |  | 97.3 | 98.6 | 96.8 | 1.28 |
| 14 | 0.56 | 1 | 0.63 | 0.37 |  | 97.0 | 98.8 | 96.8 | 1.81 |
| 15 | 0.5 | 1 | 0.63 | 0.37 |  | 97.3 | 98.5 | 96.8 | 1.20 |
| 16 | 0.5 | 1 | 0.63 | 0.37 |  | 97.1 | 98.8 | 96.8 | 1.77 |
| 17 | 0.61 | 1 | 0.74 | 0.26 |  | 97.3 | 98.6 | 96.8 | 1.28 |
| 18 | 0.53 | 1 | 0.68 | 0.32 |  | 97.3 | 98.5 | 96.8 | 1.21 |
| 19 | 0.56 | 1 | 0.63 | 0.37 |  | 97.2 | 98.8 | 96.8 | 1.59 |
| 20 | 0.5 | 1 | 0.63 | 0.37 |  | 97.1 | 98.7 | 96.8 | 1.61 |
| 21 | 0.56 | 1 | 0.68 | 0.32 |  | 97.1 | 98.7 | 96.8 | 1.59 |
| 22 | 0.5 | 1 | 0.63 | 0.37 |  | 97.3 | 98.6 | 96.8 | 1.26 |
| 23 | 0.53 | 0.95 | 0.74 | 0.21 |  | 97.0 | 98.8 | 96.8 | 1.77 |
| 24 | 0.56 | 1 | 0.63 | 0.37 |  | 97.1 | 98.7 | 96.8 | 1.61 |
| 25 | 0.56 | 1 | 0.74 | 0.26 |  | 97.1 | 98.8 | 96.8 | 1.78 |
| 26 | 0.5 | 1 | 0.68 | 0.32 |  | 96.9 | 98.7 | 96.7 | 1.75 |

*Difference between the highest and second-highest MLST scores

**Difference between the highest and second-highest ANI values
